# High-Resolution CT Following Primary Spontaneous Pneumothorax in Adolescents: Useful Tool or Wasted Radiation?

**DOI:** 10.7759/cureus.14936

**Published:** 2021-05-10

**Authors:** Sarah Stanko, Colette Oesterle, Merlin C Lowe

**Affiliations:** 1 Pediatrics, The University of Arizona, Banner-Diamond Children's Medical Center, Tucson, USA; 2 Pediatrics, El Rio Health Center, Tucson, USA; 3 Pediatrics/Hospitalist Medicine, The University of Arizona, Banner-Diamond Children's Medical Center, Tucson, USA

**Keywords:** pediatrics, primary pneumothorax, high-resolution ct scan, pulmonary bleb, surgery of the lung

## Abstract

Background

The current trend in management of first-time primary spontaneous pneumothorax (PSP) in children is to obtain a high-resolution chest computerized tomography (HRCT) scan to look for bleb/bullae disease or other forms of structural lung disease. We aimed to evaluate the significance of HRCT findings in relation to initial management strategies, and we hypothesized that these findings do not guide management.

Methods

We evaluated patients with first-time PSP in a single-institution, retrospective, longitudinal study. Data were obtained through direct chart review. The primary endpoint was the percentage of patients who underwent surgical intervention after HRCT.

Results

We identified 10 children from 10 to 17 years old from January 2013 to November 2019 who met criteria for the study. Seven out of 10 patients (70%) had HRCT after the first-time PSP during the same hospital stay. Blebs/bullae were discovered in five out of seven (71%) of those patients. Two of those five patients had subsequent surgical intervention (40%) before a recurrence. Of the three patients with blebs/bullae identified on HRCT treated without initial surgery, two had a recurrence of PSP and subsequently underwent VATS with blebectomy and pleurodesis. Among the patients without initial HRCT, there were no recurrent cases of PSP noted.

Conclusions

Our study suggests there is value in obtaining HRCT after the first time PSP, as these results can be used to guide management strategies. Further studies in pediatric PSP are needed to validate the sensitivity of HRCT in bleb detection, the predictive value of bleb disease and recurrence, and the benefits and risks of early surgical intervention.

## Introduction

Pneumothorax is defined as an accumulation of air that develops between the visceral and parietal pleura of the lung. Idiopathic or spontaneous pneumothorax occurs outside of the setting of trauma. A diagnosis of primary spontaneous pneumothorax (PSP) is given when thorough investigation reveals no underlying lung disease was causative (e.g., asthma, interstitial lung disease, cystic fibrosis, congenital pulmonary airway malformation, etc.) [1]. 

The incidence of PSP in children is reported to occur in about 4/100,000 children, with most cases occurring in adolescents 14 to 17 years of age. There is a four to one male to female predominance [2,3]. PSP classically presents in tall, thin, male adolescents. This can be due to blebs or bullae within the lung that lead to areas of weakened visceral pleura. These blebs or bullae can rupture spontaneously, leading to an air leak between the pleura. PSP can also occur without a predisposing factor. Smoking increases the risk of PSP significantly [4]. Patients present most commonly with a sudden onset of dyspnea, pleuritic chest pain, and ipsilateral shoulder pain while at rest. Interestingly, symptoms usually resolve within 24 hours, even if the pneumothorax persists.

Management strategies in children with PSP are controversial. There are limited studies and conflicting data on the subject, which contribute to the uncertainty of optimal management. Some studies report a high recurrence rate of PSP in children managed conservatively without surgical intervention [5-9]. The current trend in adolescents presenting with first-time PSP is obtaining high-resolution chest CT scans (HRCTs) to assess for blebs/bullae or other underlying disease [7,8,10-12]. This trend is supported by the findings in some studies that HRCT has high yield in identifying blebs/bullae, though it is uncertain at this time if blebs/bullae predict recurrence risk [8,10-12]. When bleb disease is identified after first-time PSP, video-assisted thoracoscopic surgery (VATS) with blebectomy and pleurodesis has become a popular intervention in an attempt to prevent recurrence [5-9].

CT is a widely used imaging modality secondary to its availability, short scan times, high-quality image production, and diagnostic capabilities in children. It also has the advantage of eliminating the need for sedation in children. The disadvantage of CT scan is the significant dose of ionizing radiation. Cumulative doses of ionizing radiation increase the lifetime risk of cancer [13]. This is especially relevant in children, since they are more radio-sensitive than adults. The radio-sensitivity in children occurs because they have longer lifespans after exposure, and also because they have more proliferating cells which tend to be more sensitive to the toxic effects of radiation. Pearce et al showed a two- to three-fold increase in the incidence of leukemia and brain tumors in people who were exposed to ionizing radiation during childhood [14].

While the risk of ionizing radiation exposure is significant, this must be balanced with the risks of a recurrent pneumothorax, subsequent hospital stays and eventual surgical intervention. We performed a retrospective longitudinal study of children with first-time PSP to determine if HRCT scan guided surgical management. Prior to our study, there have not been direct studies evaluating the utility of HRCT scan in determining the need for surgical intervention after first-time PSP. We hypothesized when HRCT is performed after first-time PSP, the results would not guide surgical management.

## Materials and methods

This study was approved by the IRB through the University of Arizona, Tucson. This retrospective longitudinal study was performed in a single institution at the Diamond Children’s Banner-University Medical Center Tucson in Tucson, Arizona. We identified children ages 10-17 years old who presented with a first-time spontaneous pneumothorax. Data were collected from the medical records from January 2013 to November 2019 using the following ICD codes: ICD 9 - 512.81 and 512.89, ICD 10 - J93.11 and J93.9 (Table 1). The unspecified pneumothorax codes largely identified non-spontaneous pneumothoraces but were included to ensure patients were not miscoded and thus missed from study. Patients with recurrent pneumothorax, prior chest surgery or other chest trauma (such as radiation therapy, ventilation injury, etc.) were excluded. We noted sex, age, BMI, laterality of pneumothorax based on CXR findings, and initial treatment of pneumothoraces (Table 2). BMI was calculated using the CDC pediatric calculator which includes age, height in inches, and weight in pounds.

**Table 1:** Descriptions of ICD codes searched. ICD: International Statistical Classification of Diseases and Related Health Problems.

ICD 9	ICD 10
512.81 Primary spontaneous pneumothorax	J93.11 Primary spontaneous pneumothorax
512.89 Other acute pneumothorax	J93.9 Pneumothorax, unspecified

**Table 2:** Patient characteristics and outcomes. ^a^Bilateral VATS blebectomy and pleurodesis; ^b^left VATS blebectomy and pleurodesis; ^c^right VATS blebectomy and pleurodesis. VATS: video-assisted thoracoscopy; BL: bilateral.

Age (years)	BMI	Sex	Laterality	Initial treatment	CT obtained	Bleb disease	Surgical intervention	Recurrence
16	18.4	Male	Left	Thoracostomy tube	Y	Y (BL apical)	N	Y^b^
14	17.7	Male	Left	Thoracostomy tube	Y	N	N	N
17	Unknown	Male	Right	Thoracostomy tube	Y	Y (BL apical)	Y^a^	N
17	18.6	Male	Bilateral	100% oxygen	Y	Y (BL apical)	Y^b^	Y^c^
14	18.8	Female	Right	100% oxygen	Y	N	N	N
16	17.1	Female	Left	Thoracostomy tube	Y	Y (BL apical)	N	N
16	Unknown	Male	Left	Thoracostomy tube	Y	Y (BL apical)	N	Y^b^
14	20	Male	Right	Observation	N	n/a	N	N
17	18.2	Female	Left	Observation	N	n/a	N	N
15	17	Male	Right	100% oxygen	N	n/a	N	N

We performed chart review to identify patient characteristics, initial treatment of patients, whether chest HRCT was obtained, presence or absence of bleb disease identified on final read of HRCT, subsequent interventions, and subsequent recurrence of pneumothorax (Table 2). In those patients who underwent HRCT, the images were reviewed by pediatric radiologists.

The primary endpoint was the rate of surgical intervention after CT chest scans in patient’s presenting after first-time PSP. The secondary endpoint was risk of recurrence with and without surgical intervention after first-time spontaneous pneumothorax.

## Results

Ten children were identified and met criteria for inclusion in the study. The average age of study patients was 15.6 years old and there was a male predominance (70% males). In two of the patients, BMI was unknown, however, for the remaining eight patients the average BMI was 18.2. These characteristics correlate with the commonly described population of patients developing PSP. There were five patients with left-sided PSP, four patients with right-sided PSP, and one patient with bilateral PSP. The initial treatments were thoracostomy tube in five patients, 100% oxygen via non-rebreather mask in three patients, and observation without intervention in two patients.

We noted that seven out of 10 patients (70%) had HRCT after first-time PSP during the same hospital stay. Of those patients who did not undergo HRCT, none required a thoracostomy tube for treatment of spontaneous pneumothorax. Of those that had HRCT, blebs/bullae were discovered in five out of seven (71%) of patients. Specifically, bilateral apical blebs were reported on the final HRCT read in all patients. Of the five patients who had blebs/bullae identified on initial HRCT, two patients had subsequent surgical intervention immediately after this finding (40%), and an additional two patients had surgical intervention after recurrence of pneumothorax. Among the 70% who had HRCT performed, 28% had surgical intervention initially, and 57% had surgical intervention in total (both after first-time PSP and after recurrence). Of the three patients who had positive blebs/bullae on HRCT but did not undergo initial surgical intervention, all had thoracostomy tubes placed as initial treatment. Of those three patients, two had recurrence of PSP and underwent VATS with blebectomy and pleurodesis after recurrence (recurrence rate of positive bleb/bullae disease = 66%). Among the patients who did not have initial HRCT, there was no recurrence of PSP noted. Table 3 summarizes these results.

**Table 3:** Summary of findings. BL: bilateral; HRCT: high-resolution computed tomography; PSP: primary spontaneous pneumothorax; PTX: pneumothorax.

Patient group	% of patients
First time PSP with HRCT done	70%
Bilateral blebs identified	71%
BL blebs with initial surgery	40%
BL blebs with surgery after recurrence	40%
BL blebs with surgery (total)	80%
HRCT	
HRCT with initial surgery	28%
HRCT with surgery at any point	57%
Recurrence rate of PTX in patient with BL blebs without initial surgery	66%
First time PSP without HRCT	0% needed thoracostomy tube, 0% recurrence of PTX

## Discussion

Primary spontaneous pneumothorax is rare in children, and significant debate remains about optimal management strategies, use of HRCT after first-time occurrence, timing of surgical intervention, and recurrence risk. Our hypothesis at the beginning of this study was that HRCT would not identify a high proportion of patients with bleb disease (and as such, not require further surgical intervention) suggesting HRCT may not be needed after the first PSP; however, our results show that a high percentage of patients with first-time PSP did have findings on HRCT leading to surgical intervention.

The yield of HRCT in identifying bleb/bullae disease in children after first-time PSP when compared to intraoperative identification is still uncertain at this time. In our study, there was a 100% yield of HRCT for intraoperative bleb detection, though our sample size is small and included no occurrences of operative management without preceding HRCT revealing bleb disease. Similarly, Nathan, et al. found a sensitivity of 75% for CT scan identification of blebs confirmed intraoperatively among 25 patients under the age of 18 years old [8]. In contrast, Laituri et al. evaluated 10- to 23-year-old patients who had preoperative chest CT scans prior to VATS, and the sensitivity of CT scan for identifying blebs that were found intraoperatively was only 36% [15]. Future studies are needed to determine whether younger patients have increased sensitivity of CT scan for bleb identification. Additionally, studies aimed at establishing sensitivity of HRCT for identifying patients with bilateral apical involvement of blebs could provide evidence of a higher risk group likely to benefit from early CT to better guide operative management.

HRCT scan may be useful in guiding surgical management with the ultimate goal of reducing recurrence rate. In our study, 40% of patients with bleb/bullae disease identified on HRCT underwent surgery during the initial hospital admission. An additional two patients with bilateral apical bleb disease identified on HRCT experienced recurrence of symptoms, and subsequently underwent VATS procedures, ultimately resulting in 80% of those with identified bleb disease on initial HRCT requiring surgical intervention overall. Among the 70% who had HRCT performed, 28% had surgical intervention initially, and 57% had surgical intervention in total (both after first-time PSP and recurrence). 

Interestingly, all patients identified by HRCT to have bleb disease showed bilateral apical involvement, indicating a potential subset of patients undergoing spontaneous pneumothorax at higher risk physiologically for both recurrence and severity of symptoms. Patients found to have bilateral bleb disease may be more likely to benefit from aggressive surgical management, as our results indicate that 80% of these patients ultimately required surgical intervention in this pilot study. Further research is needed to better define clinical characteristics leading to the decision to obtain HRCT in these patients initially. Correlation between severity of presentation and initial radiograph findings with the clinician’s decision to obtain HRCT may indicate some evidence of risk stratification occurring in clinical decision making in practice. This would be supported by the finding that all our patients who needed a chest tube subsequently underwent HRCT and four out of five (80%) of those patients had bleb disease with ultimate surgical intervention.

Many studies have found a decrease in recurrence rate with initial operative management versus non-operative management of children and adolescents with first-time PSP [5-9]. There is uncertainty about bleb disease predicting recurrence in these studies. Our study found the recurrence rate of patients with blebs visualized on HRCT and treated conservatively was 66%, while those who did not undergo HRCT and were treated conservatively had a 0% recurrence rate. In Segiuer-Lipszyc et al. the recurrence rate among conservatively treated patients was 50%, and recurrence rate was significantly lower following surgical intervention [6]. In contrast to our findings, they found that recurrence rates in cases with and without blebs on CT were comparable [6]. Similarly, in Soler et al., recurrence rate was 45% in the operative group compared to 14% in the non-operative group. They found that a negative CT scan for subpleural blebs did not predict recurrence [7]. Lopez et al. showed 40% recurrence rate for those treated nonoperatively and noted that initial nonoperative management resulted in more total hospital days [5]. The low recurrence rates our study revealed in patients not evaluated by HRCT may indicate that these were mild cases, with clinical features indicating low severity. Most of the patients who did not undergo HRCT were treated only with observation, or supplemental oxygen, with no patients in this group requiring thoracostomy tube treatment. We cannot determine exactly why these patients did not undergo HRCT, though it was likely due to less severe physical symptoms at presentation. Again, this may point to potential for differentiation of presenting patients with spontaneous pneumothorax into high and low-risk groups. Further studies are needed to examine the clinical features evaluated when deciding to obtain HRCT.

Adult British Thoracic Society (BTS) guidelines support observation alone without needle aspiration in patients with small (<2 cm) PSP and/or not breathless [16]. In our study, the patients who did not undergo HRCT were presumed to have very mild symptoms, and therefore the BTS guidelines could only be applied to this subset of patients. The adult BTS guidelines note that CT scanning is the gold standard in detection of small pneumothoraces and in size estimation, and also helpful in identifying underlying lung pathology, but practical constraints preclude its general use as the initial diagnostic modality. Surgical consultation is not recommended until secondary spontaneous pneumothorax occurs or unless persistent air leak greater than 24 hours with other methods (chest tube, etc). Our results suggest that these specific recommendations may not apply to children, based on our findings of high recurrence risk requiring surgical intervention in children with bilateral apical blebs identified on HRCT after PSP. The adult BTS guidelines do not take into consideration the relationship between the presence of bullae and risk of recurrence in children. Similarly, Soccorso et al. comments that adult BTS guidelines aren’t applicable to children with large PSP, considering the high recurrence rate (50%) if contralateral asymptomatic bullae were present [11].

Our study is limited by small sample size and serves best as a pilot study for further investigation. Three patients did not have an HRCT performed, and their management may have been different if they had this imaging. We do not know if they had bleb disease and thus we cannot determine if these three patients would support or refute the utility of an HRCT in PSP. Limitations to this study include loss of data regarding whether additional care was received for pneumothoraces at another facility. Despite these limitations, we believe the findings support further research to better characterize the utility of HRCT in first-time PSP.

## Conclusions

Spontaneous pneumothorax in adolescents is rare and, given the overall limited number of studies and conflicting findings of current studies, there remains debate about optimal management strategies. The concern about risks in exposing pediatric patients to ionizing radiation has led to differing opinions about obtaining an HRCT after first-time spontaneous pneumothorax and there is no widely accepted protocol or algorithm to guide these clinical situations in pediatrics. Factors that need to be further explored include yield of HRCT in identifying blebs, presence of blebs in predicting recurrence, and other clinical factors at presentation that may indicate higher risk for recurrence with conservative management. In our retrospective observational study, in contrast to our initial hypothesis that HRCT would not routinely identify findings suggesting high risk of pneumothorax recurrence, we found that a high percentage of patients who underwent HRCT after first-time spontaneous pneumothorax did have bleb disease and that said findings did have a high risk of recurrent pneumothorax. These findings may aid in guiding the need for surgical intervention, as 80% of patients with bleb disease in our study did undergo surgery at some point. As a pilot study, we illustrate that further studies in pediatric first-time spontaneous pneumothorax could help to develop a risk stratification at initial presentation to help guide the decision to obtain HRCT and identify those who may benefit from early surgical intervention.
